# Dynamic and distinct physiological responses by a soil bacterium promote survival along a desiccation continuum

**DOI:** 10.1128/mbio.01903-26

**Published:** 2026-09-01

**Authors:** Jarek V. Kwiecinski, Georgia R. Squyres, Dani Or, Dianne K. Newman

**Affiliations:** 1 Division of Geological and Planetary Sciences, California Institute of Technology 224033 , Pasadena, California, USA; 2 Division of Biology and Biological Engineering, California Institute of Technology 6469 , Pasadena, California, USA; 3 Department of Environmental Systems Science, Swiss Federal Institute of Technology, Zürich, Switzerland; 4 Department of Civil and Environmental Engineering, University of Nevada, Reno, Nevada, USA; Georgia Institute of Technology, Atlanta, Georgia, USA

**Keywords:** osmolytes, desiccation, starvation, dormancy, soil

## Abstract

Soil bacteria play a central role in global biogeochemical cycles and are critical for soil health and agricultural productivity. The dynamic nature of soil hydration status affects bacterial habitats by changing the energy state of soil water and disrupting aqueous connections critical for nutrient diffusion. To study how soil bacteria respond to desiccation, we used the rhizobacterium *Pseudomonas synxantha* 2-79 as a model organism and quantified its response to co-occurring water and nutrient limitations at the single-cell level. We hypothesized that the relative importance of osmolyte synthesis and starvation responses to desiccation tolerance is context dependent, with the optimal strategy determined by the trajectory of nutrient and water deprivation. We constructed a transcriptional reporter to track *P. synxantha*’s expression of biosynthesis genes for the osmolyte N-acetylglutaminylglutamine amide (NAGGN) and collected extensive single-cell growth rate, cell size, and reporter expression data through experiments that mimicked different rates and extents of soil drying. Only actively growing cells responded to an osmotic shock by synthesizing NAGGN; this response was not observed for pre-starved bacteria. Despite the lack of osmolyte NAGGN synthesis, prior starvation enhanced *P. synxantha’*s ability to recover from osmotic stress once water and nutrients were restored. In line with our observation that prior starvation prevented cell lysis upon rewetting, starved cells had more rigid membranes. Together, our results indicate that diverse cellular properties contribute to soil bacterial desiccation tolerance, whose relative response and fitness are tuned to different challenges imposed by soil drying dynamics.

**IMPORTANCE:**

Soil bacteria are critical to agriculture, but it is unclear how these organisms respond to desiccation, a common and worsening stress. Desiccation both dehydrates bacterial cells and eliminates the liquid water connections between soil pores that bacteria use to access nutrients. We studied how a model soil bacterium responds to (co)-occurring starvation and water stress at the single-cell level, focusing on osmolyte synthesis and physiological adjustments that take place under starvation. We describe the desiccation and regrowth trajectories in these conditions at single-cell resolution. We find that starvation restricts synthesis of a dipeptide osmolyte but rigidifies the membrane, enabling bacteria to withstand more severe water stress. Distinct cellular factors thus contribute differentially to desiccation tolerance along a drying trajectory.

## INTRODUCTION

As Earth’s largest biome, drylands are home to approximately 2 billion people and occupy approximately 41% of the planet’s land area (1). Within these ecosystems, soil bacteria play a vital role in nutrient cycling, promote plant growth, and can confer enhanced drought tolerance to crops (2, 3). During dry periods, soil bacteria lose access to nutrients and experience decreases in soil matric water potential (Ψ), a quantity typically expressed with units of pressure (i.e. energy per volume) that captures the effects of capillarity and adhesion on the chemical potential of pore water (4). Upon rewetting, nutrient access is restored, and cells become rehydrated; this drives a respiratory pulse known as the Birch effect (5).

**Fig 1:**
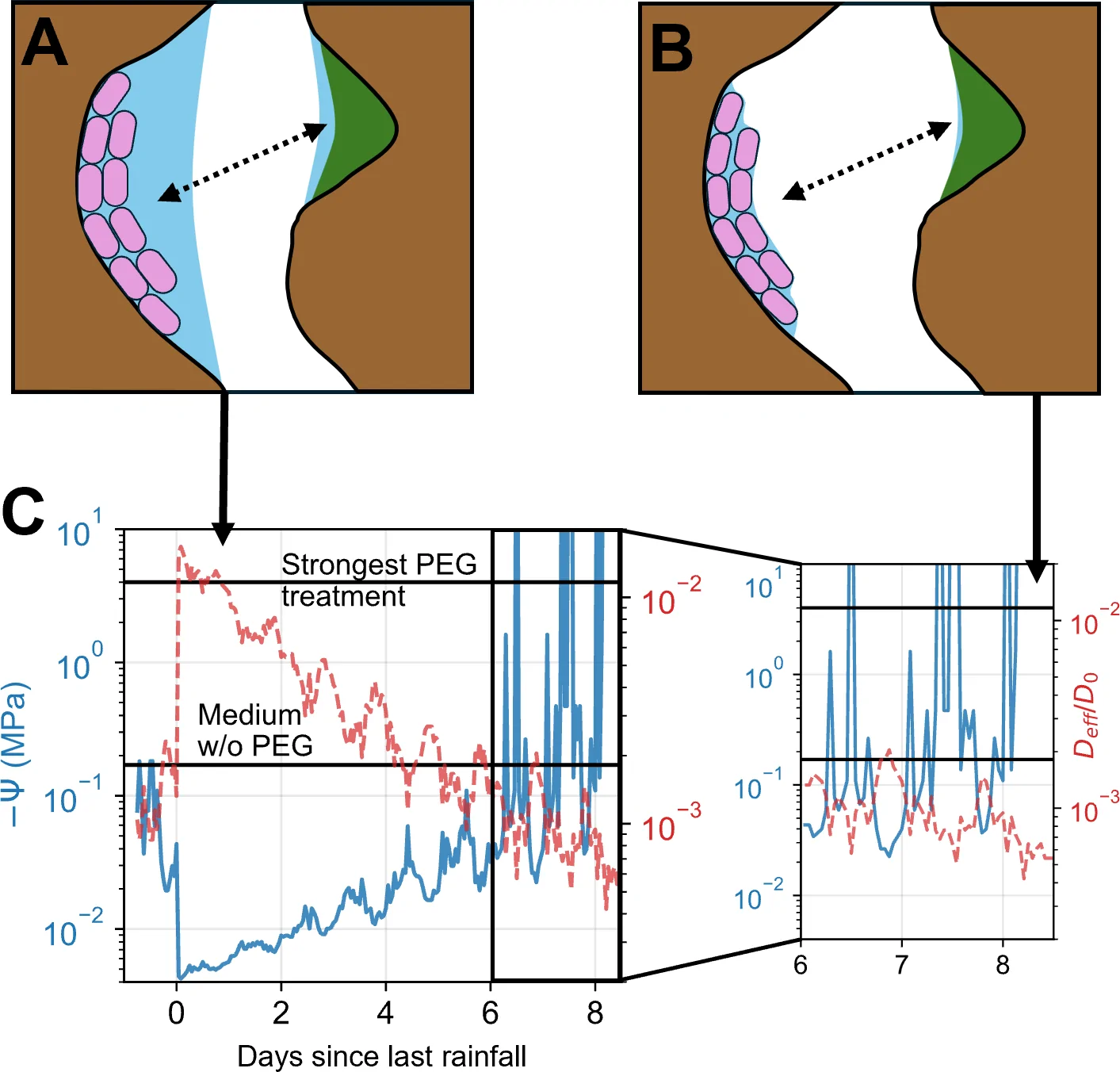
Solute diffusivity is reduced even in relatively wet soils. The cartoons show soil pores in different hydration states. In panel **A**, bacteria (purple rods) are well hydrated but lack a hydraulic connection to their substrate (green), meaning that they have no means to access nutrients from their surroundings. Panel **B** shows the same soil pore in a more advanced stage of drying at which bacteria begin to desiccate. Panel **C** shows how these dynamics play out in a real soil, supporting the notion that starvation precedes desiccation. Soil moisture data were taken from a site administered by the National Water and Climate Center at the Jornada Experimental Range, New Mexico (14). The data show soil drying after a 2.7 cm rain event during the 2025 North American Monsoon. We computed the bulk-averaged effective diffusivity of dissolved nutrients from this data according to the equation in reference 15. Even immediately after rainfall, the effective diffusivity was only ~1% of solute diffusivity in bulk water (red dashed line). This rain event did not saturate the soil, so there was limited hydraulic connectivity between any two points. Many soil microsites may have looked like the cartoon in panel **A**. The water potential at this time, as estimated from soil moisture using a van Genuchten retention curve (16), is less negative than even the culture medium we used as a no-water-stress control (medium without polyethylene glycol (PEG), Ψ = −0.17 MPa). The retention curve was parameterized using values provided for the field site, where the soil was 93% sand. Six days after rainfall, the soil had dried to the water potential of our culture medium, at which point it began to rapidly fluctuate with diurnal variations in relative humidity at the field site. These fluctuations repeatedly drove the water potential below −4 MPa, our most intense experimental condition, within the hourly sampling interval (see inset). Together, these data show that solute diffusion is generally restricted in unsaturated soils, and that the nonlinear relationship between water potential and soil moisture can drive rapid variations in water availability as soil dries.

Although studies of water stress often focus on its osmotic effects, in soil, fluctuations in the availability of water cause cells to experience both nutritional and biophysical stresses. While bacterial responses to water loss alone have been characterized (6), we know less about how bacteria adapt to the tandem water and nutrient challenges that occur during drying in soil. Desiccation proceeds along a continuum (Fig. 1). In a saturated soil, nutrients can freely diffuse through pore water, and bacteria are in osmotic equilibrium with their environment. As soils begin to dry, nutrient diffusivity drops dramatically (Fig. 1A) (7). Nutrient diffusivity is a critical variable for soil bacteria because they inhabit thin liquid films that, under even modestly dry conditions, are too small to support motility (8). Diffusion is thus the primary means by which bacteria access dissolved substrates. As drying proceeds, liquid films become thinner, and aqueous connections between pores are disrupted, increasing the diffusion path length between bacteria and nutrient sources (9). Consequently, effective solute diffusivity decreases significantly, perhaps even at relatively mild water potentials (10), which are easily tolerated by most bacteria (11, 12). Eventually, the soil matric water potential decreases below the water potential of the cytoplasm, causing water to leave the cell (13) (Fig. 1B). At more extreme matric potentials that threaten cellular integrity and viability, nutrient transport essentially stops altogether (4, 10) (Fig. 1C).

How do cells cope along this dynamic desiccation trajectory? In water-replete environments where nutrients are readily available, it is well established that bacteria adapt to water potential disequilibria by synthesizing osmolytes that allow them to retain cytoplasmic water while protecting proteins from damage (6, 17). Yet, this strategy requires a significant resource investment that may be impractical within the diffusion-limited soil environment. This challenge is made especially acute in dry soils, where the water potential can decrease to levels at which osmolytes would have to account for essentially all of microbial biomass to prevent dehydration (18). Therefore, it seems unlikely that bacteria could survive desiccation by using osmolytes to retain all of their cytoplasmic water. Osmolytes can protect cellular machinery from damage at concentrations lower than what would be required to prevent water loss (19). However, the starvation that accompanies desiccation causes myriad other changes to the cell that may also promote desiccation tolerance.

How could starvation, which limits the resources available for osmolyte synthesis, promote desiccation tolerance? At the transcriptional level, nutrient depletion represses oxidative metabolism, as well as nucleotide and rRNA biosynthesis, limiting oxidative stress and slowing overall transcription and translation rates (20). Reactive oxygen species accumulate under desiccation when the electron transport chain is disrupted due to membrane damage (21), so repression of oxidative metabolism under starvation may protect desiccated cells from oxidative damage. Starvation also upregulates genes that promote ribosome hibernation, inhibit DNA replication, and regulate carbon storage (22). Consistent with these observations, prior starvation has been shown to help *Escherichia coli* better survive an osmotic shock (23), and nitrogen deprivation can enhance the survival of some cyanobacteria through desiccation (24, 25). The protective effects of nutrient deprivation may thus represent a key component of soil bacterial water stress survival that transcends osmolyte synthesis.

Starvation and water limitation also have similar biophysical effects on bacterial cells that motivate the study of these stresses at the single-cell level. Sudden nutrient or water deprivation both cause the cytoplasm to shrink due to water loss (26). Overall cell size further decreases as starvation progresses due to resource limitations slowing cell expansion and regulatory links between cell division and metabolism (27). Starvation and desiccation also reshape cell membranes: both stresses alter membrane lipid composition (28, 29), while drying causes lipid phase changes that make the membrane vulnerable to permeabilization or rupture upon rewetting (30). It is thus interesting to consider how the mechanisms and timing of cell death are shaped by combinations of starvation and desiccation given their ubiquitous co-occurrence in the environment.

Accordingly, we set out to investigate how nutrient and water limitations jointly shape the bacterial desiccation response at single-cell resolution. We chose to work with *Pseudomonas synxantha* 2-79, a model soil bacterium whose molecular and cellular responses to desiccation can be studied using live microscopy. In a system that enables precise environmental control, we collected single-cell measurements of cell size, growth rate, osmolyte gene expression, and general protein synthesis during starvation and water stress. By imposing starvation and water stress independently or in combination, we disentangled the effects of these components of desiccation on *P. synxantha*’s physiology. We also chose treatments that simulated different soil drying trajectories. We hypothesized that the relative importance of osmolyte synthesis and starvation responses to desiccation tolerance is context-dependent, with the optimal strategy determined by the dynamics of nutrient and water deprivation.

## RESULTS

We considered three scenarios to probe the effects of starvation and water stress in isolation and in tandem. First, we conducted experiments where we reduced the water potential in the presence of nutrients. Second, we simultaneously removed nutrients and lowered the water potential, simulating how bacteria become disconnected from their nutrient supply under desiccation. Finally, we starved bacteria before lowering the water potential, mimicking cases where nutrient diffusivity decreases before water availability restricts microbial activity, a possibility illustrated in Fig. 1. In all cases, we mimicked rewetting at the end of the experiment by restoring water and nutrients and tracked cellular responses using a transcriptional reporter.

### Developing a transcriptional reporter to monitor osmolyte biosynthesis gene expression

**Fig 2:**
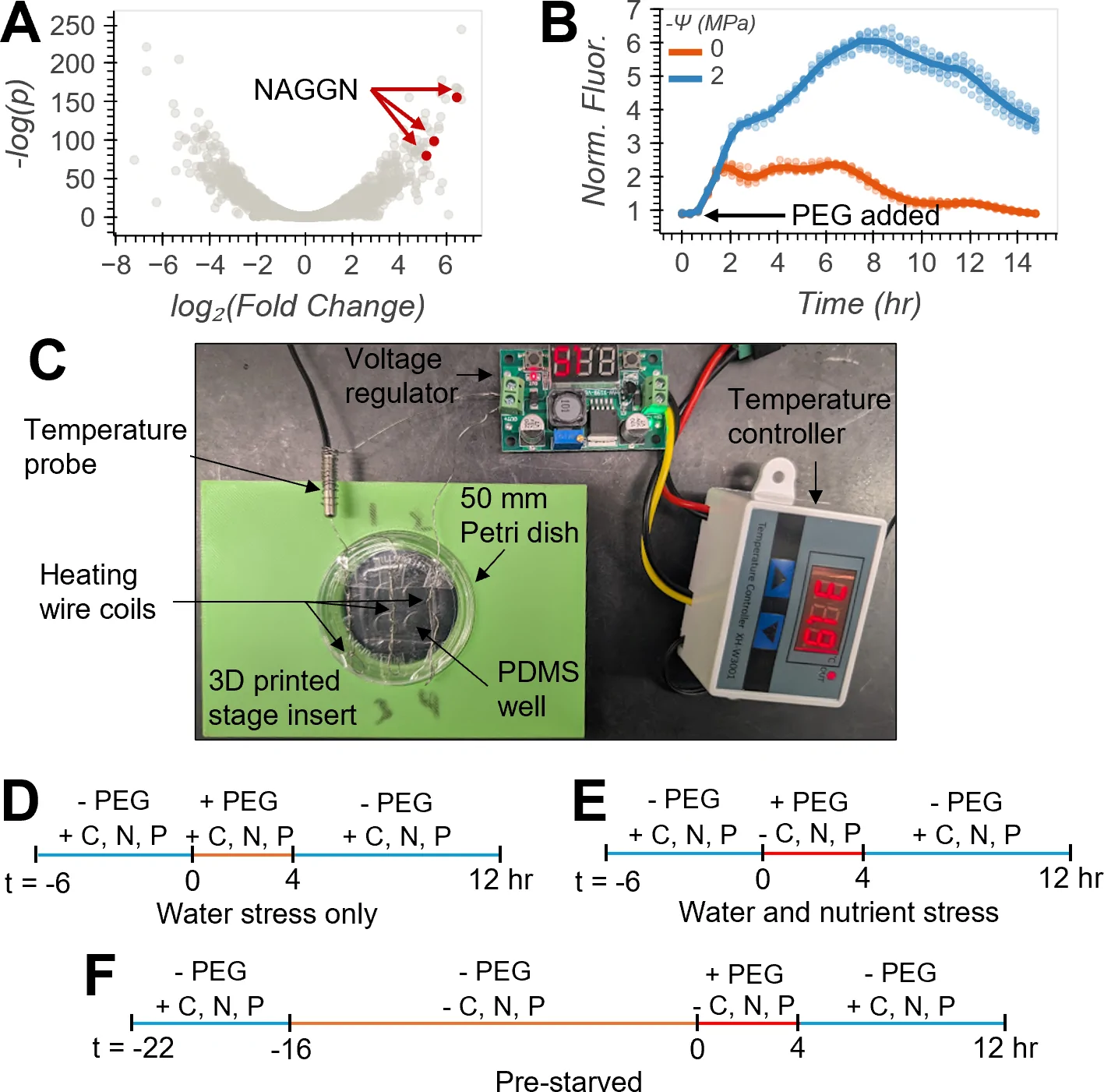
RNA-seq results, reporter verification, and setup for microscopy experiments. (**A**) Decreasing water potential with PEG alters *P. synxantha* gene expression. Genes with positive fold change values were upregulated upon exposure to PEG. Genes with smaller *P* values [higher −log(*P*)] were covered by more sequencing reads or had larger fold changes, increasing confidence that these genes were in fact differentially expressed. N-acetyl glutaminylglutamine amide (NAGGN) biosynthesis genes were the most upregulated genes for osmolyte synthesis and are highlighted in red. (**B**) The ratio of mNeonGreen to mApple fluorescence from our transcriptional reporter for osmolyte synthesis. mNeonGreen was expressed under control of a putative NAGGN promoter and mApple was expressed under control of the constitutive P_nptII_ promoter. Colors indicate the water potential the growth medium was adjusted to at *t* = 1 h using PEG 1000. Points indicate the normalized fluorescence in individual replicates in a 96-well plate, and lines show the mean. (**C**) Photograph of the experimental setup used to impose water and nutrient shifts over time and monitor cell behavior microscopically. Cells were grown in polydimethylsiloxane (PDMS) wells within a 50 mm glass-bottomed petri dish affixed with a heated lid used to prevent fogging. (**D–F**) Timelines showing when PEG and nutrients were added and removed for each of our experimental conditions, which included different combinations of water and nutrient stress.

To test the hypothesis that the osmolyte synthesis response to water stress is condition-dependent, we created a transcriptional reporter to monitor osmolyte biosynthesis gene expression. The first step in designing this reporter was to identify the osmolyte biosynthesis genes that *P. synxantha* upregulates the most under water stress. We therefore conducted an RNA-seq experiment in which we transferred a mid-exponential phase culture of wild-type *P. synxantha* to a new medium with the same nutrient composition but augmented with 280 g/L polyethylene glycol (PEG 8000, average molecular weight = 8,000 Da) to impose water stress. This medium was designed to mimic the average elemental composition of soil microbial biomass (31), and with PEG added, it had a water potential Ψ = −2.66 MPa. PEG is used in studies of plant and bacterial water stress in place of salts because it is nonionic and therefore has no direct effect on membrane potential or ion homeostasis (29). Even though PEG of this size can be imported and metabolized by some pseudomonads (32), it has distinct effects on cellular ultrastructure and membrane composition from more permeating solutes like NaCl or PEG 200. These changes better match what happens to cells under desiccation (29). Following this treatment, we extracted, purified, and sequenced RNA before processing reads to evaluate differential gene expression between control and water stress treatments (Fig. 2A).

The most upregulated osmolyte synthesis genes were those associated with N-acetyl glutaminylglutamine amide (NAGGN) biosynthesis (*ggnAB* and a putative peptidase, *P. synxantha* 2-79 locus tags C4K02_RS19705, RS19710, and RS19715, log_2_ fold changes of 5.2, 5.5, and 6.5). First discovered in *Sinorhizobium melitoti* (33), this dipeptide osmolyte is widely used by pseudomonads (34), especially the epiphytic plant pathogen *Pseudomonas syringae* B728a (35). NAGGN production is known to be particularly resource intensive, and NAGGN synthesis can even harm some bacteria under osmotic stress because its production is so costly (35, 36). This made it an especially compelling target for our experiments, given our goal to investigate how starvation shapes *P. synxantha’s* water stress response.

Both of *P. synxantha*’s biosynthesis pathways for the osmolyte trehalose (37), one using *treS* and the other using *treY* and *treZ*, were also upregulated in response to PEG addition. Furthermore, *P. synxantha* upregulated genes for exogenous osmolyte transporters, including the ATP-dependent glycine betaine transporter YehZYXW. Restricted solute diffusion in dry soil would limit the transport of external osmolytes to bacteria, so the accumulation of exogenous osmolytes may also be challenging under desiccation. Finally, of *P. synxantha’s* eight mechanosensitive channels, two were upregulated and one was downregulated (|log_2_ fold change| > 1, *P* < 0.05) following PEG addition. These proteins allow for rapid solute release following a hypoosmotic shock (38) and are thus likely important to *P. synxantha*’s survival following rewetting. Detailed RNA-seq results for these genes are available in Table S1 and complete RNA-seq data are provided in Data set S1.

To enable the detailed microscopic observations described below, we chose to monitor osmolyte gene expression using a transcriptional reporter for NAGGN biosynthesis. This targeted reporter could not capture the effects of nutrient and water stress on other osmolytes or water stress response pathways. However, it allowed us to screen more conditions with a greater temporal resolution than would be possible using an untargeted approach.

To design a transcriptional reporter for NAGGN biosynthesis (see Fig. S1), we first introduced the fluorescent protein mApple to a neutral chromosomal site downstream of a constitutive promoter (39). This fluorescence channel provided us with data on *P. synxantha*’s general ability to perform translation under the treatments we imposed. We transformed this strain with a plasmid harboring a putative promoter sequence for NAGGN biosynthesis (40) upstream of the gene for the fluorescent protein mNeonGreen. Attempts to put both the constitutive and NAGGN reporter constructs on the plasmid or both in the chromosome were unsuccessful, so we proceeded with this configuration. Having both constructs in the same strain allowed us to compute the ratio between mNeonGreen and mApple fluorescence (hereafter normalized fluorescence), a metric that accounts for factors beyond changes in gene expression that would affect reporter fluorescence. These factors include treatment effects on overall transcription and translation ability, fluorescent protein brightness, or changes in growth rate that would alter the rate at which fluorescent proteins are diluted into more biomass. Finally, we verified that this completed reporter construct was responsive to water stress by exposing exponential phase reporter cells to a water potential Ψ = −2 MPa using PEG 1000 in a 96-well plate. Within 2 h of exposure, the normalized fluorescence exceeded that of reporter cells that did not experience water stress (no PEG added, Ψ = 0 MPa in Fig. 2B). PEG 1000 was used for this and future assays because of difficulties manipulating viscous PEG 8000 solutions. Though it is likely more membrane permeable than PEG 8000, PEG 1000 is still a larger molecule than solutes like sucrose that are commonly used for osmotic stress experiments. It is also uncharged, again avoiding confounding effects on ion homeostasis that are not relevant to the unsaturated environments represented by our experiments.

After verifying our reporter’s responsiveness to water stress via PEG addition, we monitored cell size, growth, and reporter activity over different conditions. We conducted these experiments using an apparatus adapted from reference 41 consisting of a glass-bottomed Petri dish with four polydimethylsiloxane (PDMS) wells (Fig. 2C). The bottom of the wells was coated with an adhesive that held cells in place even when removing and replacing the fluid in the wells. This allowed us to monitor cell behavior through a series of nutrient and water potential changes, outlined in Fig. 2D through F. In all cases, exponential phase reporter cells were first adhered to the bottom of the wells and allowed to adjust to the surface for 6 h. Thereafter, we switched culture conditions by removing and replacing the full volume of the medium in the wells, changing the nutrient and PEG content as indicated. During our experiments, we collected single-cell phase-contrast and fluorescence microscopy images, segmented them to locate the boundaries of cells, and extracted single-cell morphological data, growth rates, and fluorescence intensities. Experiments were done in biological duplicate, so we studied how two different populations of cells responded to the same treatments. In each replicate, there were roughly 45–470 segmentation masks containing single cells or groups of cells present throughout the PEG treatments, with numbers varying between conditions. See Materials and Methods and Fig. S5 and S6 for details.

### Response of *P. synxantha* to reduced water potential in the presence of nutrients

We first observed *P. synxantha*’s cellular response to a decrease in water potential in the presence of nutrients (Fig. 2D). This condition represents the most well-studied case of bacterial water stress in which changes in medium water potential are decoupled from nutrient availability. It thus served as a point of comparison to understand how nutrient deprivation drives deviations from the bacterial response to water stress alone. We tested water potentials of Ψ = 0, −1, −2, and −4 MPa, specified by PEG addition to the targeted values. Soil can dry beyond these potentials, but our Ψ = −4 MPa treatment is severe enough to restrict the growth and respiration of diverse soil bacteria (12).

We recognize that soils often dry gradually, with rates varying due to atmospheric demand, antecedent water content, and soil depth (42). However, due to the highly nonlinear relationship between water content and water potential in soil (16), we expect that as soil dries, small changes in water content lost to evaporation can induce large changes in water potential, as illustrated in Fig. 1C. Our experiments, which also impose sudden changes in water potential, capture a subset of the drying trajectory outlined in this figure, focusing on state-altering transitions where a small loss of water to evaporation may induce large changes in water potential.

**Fig 3:**
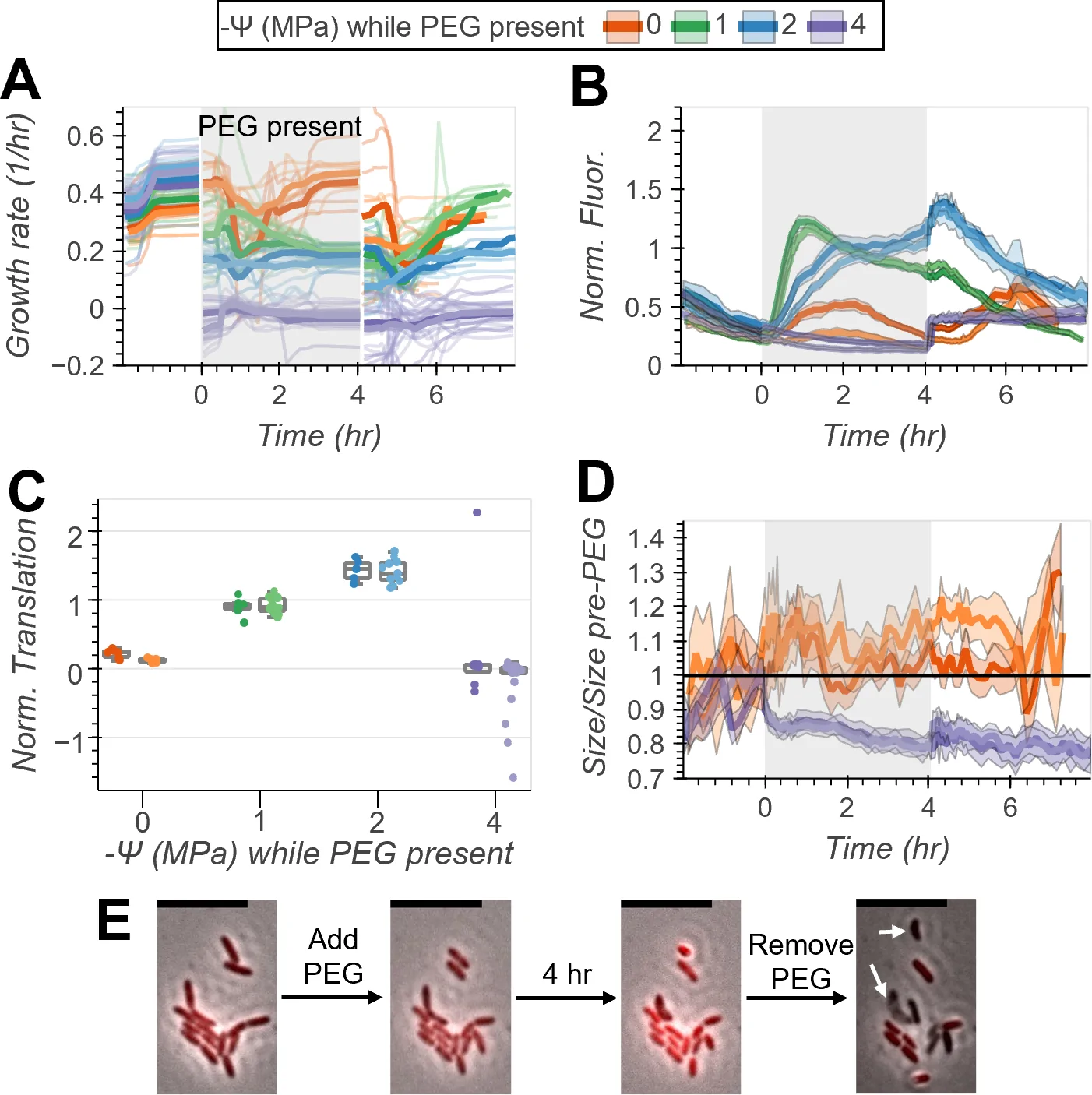
Cellular responses to water stress vary with the severity of the stress in *P. synxantha*. (**A**) Growth rate before, during, and after PEG exposure. PEG was present for the time range shaded in gray. Bold lines show the mean growth rate across all microcolonies tracked to measure growth (see Materials and Methods). Faint lines show growth rates for individual microcolonies. The number of microcolonies measured at each time point is displayed in Fig. S7. Colors indicate different water potentials during PEG exposure, with darker and lighter shades distinguishing between replicate experiments. (**B**) NAGGN reporter fluorescence normalized by constitutive fluorescence. Lines represent the mean normalized fluorescence across all segmentation masks and ranges show 95% confidence intervals. The number of segmentation masks used to compute the mean fluorescence can be found in Fig. S5. During PEG exposure, there was an average of 46–204 masks present, varying between water potentials and replicate experiments. (**C**) The ratio of total estimated mNeonGreen to mApple translation calculated using a model that accounts for photobleaching and growth dilution effects (see Materials and Methods). Points represent microcolonies. Higher values of this ratio correspond to greater overall NAGGN gene expression. Some values are negative for the Ψ = -4 MPa cells because values of both integrated mNeonGreen and mApple translation were near zero (see Fig. 6), and the model does not account for fluorescent protein leakage or degradation. (**D**) Size of reporter cells in the Ψ = 0 and -4 MPa treatments, normalized to their mean size in the 30 min leading up to PEG addition. Mean sizes displayed as lines were computed for segmentation masks filtered to contain only one cell (see Materials and Methods). The number of filtered masks used to compute mean sizes can be found in Fig. S6. During PEG exposure, there was an average of 33–165 cells present, varying between water potentials and replicate experiments. (**E**) Images of one microcolony of reporter cells immediately before PEG addition, immediately after the water potential was lowered to Ψ = -4 MPa, after 4 h of exposure to this water potential, and immediately after PEG was removed. The phase contrast and control fluorescence layers are overlaid to show changes in cell morphology and membrane permeabilization leading to loss of mApple upon PEG removal. White arrows in the last image point to cells that lysed or were permeabilized. Scale bars are 10 µm. Red fluorescence data are all displayed on the same intensity scale.

When the water potential was lowered to Ψ = −1 MPa, cells continued growing, mounted a robust osmolyte synthesis response, and shrank very little despite the imposed hyperosmotic shock (Fig. S2A). However, cells immediately reduced their growth rate to roughly two-thirds the value observed before PEG addition (Fig. 3A). NAGGN biosynthesis genes were upregulated almost immediately, and both the normalized (Fig. 3B) and mNeonGreen fluorescence (Fig. S2C) reached an initial peak after 1 h. The normalized signal began decreasing before PEG was removed, indicating that the rate of NAGGN biosynthesis gene expression had begun to decrease relative to the rate of constitutive mApple biosynthesis. The normalized reporter signal decreased more rapidly once PEG was removed. This is due to a combination of decreased NAGGN gene expression, as expected with the removal of water stress, and recovering cell growth rates (see increased growth rate after *t* = 4 h in Fig. 3A), increasing the rate at which the remaining mNeonGreen was diluted into more biomass.

*P. synxantha* also tolerated a shift to Ψ = −2 MPa with only modest changes to cell morphology and growth. Though this treatment caused a greater initial decline in growth rate, cells from both the Ψ = −1 and −2 MPa treatments converged on a similar growth rate equal to about half that observed in the untreated cells by the end of PEG exposure (Fig. 3A). Cells shrank only slightly and regained their original size within 1 h of PEG addition (Fig. S2B), by which time NAGGN biosynthesis gene expression had already begun (Fig. 3B and Fig. S2C). Reporter fluorescence began to increase later than in the Ψ = −1 MPa treatment, suggesting a lag between the onset of water stress and osmolyte gene expression. Both the normalized (Fig. 3B) and mNeonGreen fluorescence (Fig. S2C) continued increasing until PEG was removed, so even though NAGGN biosynthesis gene expression began later, it continued for longer. We estimated total fluorescent protein production during the PEG treatment, applying a model that accounted for protein maturation kinetics, photobleaching, and dilution via growth (see Materials and Methods). The ratio of estimated mNeonGreen to mApple translation was highest in the Ψ = −2 MPa treatment, so when accounting for differences in overall translation capacity, this water potential prompted the most osmolyte gene expression (Fig. 3C). When nutrients are available, the milder Ψ = −1 and −2 MPa conditions are clearly within *P. synxantha*’s ability to osmoregulate, and NAGGN biosynthesis represents a part of *P. synxantha*’s response to a modest shift in water availability.

However, cells exposed to a water potential of Ψ = −4 MPa followed a different trajectory. PEG addition immediately halted their growth, and growth did not resume even after PEG was removed (Fig. 3A). The cells also shrank substantially, losing up to 15% of their original size within 1 h of PEG addition (Fig. 3D). Shrinkage continued at a slower rate for the rest of the 4 h PEG treatment, and the cells ended at ∼80% of their original size before PEG was removed. Cells that survived PEG removal briefly increased in size before they shrank once again (see small increase at *t* = 4 h in Fig. 3D), and no detectable NAGGN gene expression occurred at any point in the experiment (Fig. 3B and Fig. S2C). This is despite the fact that translation was still possible, as indicated by the increase in control fluorescence (Fig. S2D) reflecting ongoing mApple translation during PEG exposure (purple points in Fig. 6A, water stress only experiment). However, whatever translation occurs under these conditions is insufficient to recover cytoplasmic water during PEG exposure or to prevent widespread cell lysis upon PEG removal. Lysed cells were visible in images taken immediately after PEG removal (Fig. 3E). Other cells were permeabilized and lost fluorescence, consistent with fluorescent proteins leaking into their surroundings. This was evident in the control fluorescence data, with sharp drops in fluorescence occurring immediately after PEG was removed (Fig. S2D). Exponentially growing, unadapted cells are thus not equipped to survive rapid, large shifts in water potential.

### Response of *P. synxantha* to simultaneous removal of nutrients and water

After establishing how *P. synxantha* responds to water stress in the presence of ample nutrients, we considered the opposite extreme in which cells lose access to nutrients and begin to dehydrate simultaneously. We observed *P. synxantha*’s cellular responses to a decrease in water potential and the simultaneous removal of nitrogen, phosphorus, and all carbon sources except for PEG. This condition provides no window for adaptation prior to the onset of tandem water and nutrient stress and therefore represents a worst-case scenario for soil bacteria. We again performed duplicate experiments, with one difference: the inocula for each experiment began at different growth rates prior to the onset of water and nutrient stress (see growth rates where *t* < 0 in Fig. S3A and B). This fortuitous difference revealed nuanced and interesting responses.

**Fig 4:**
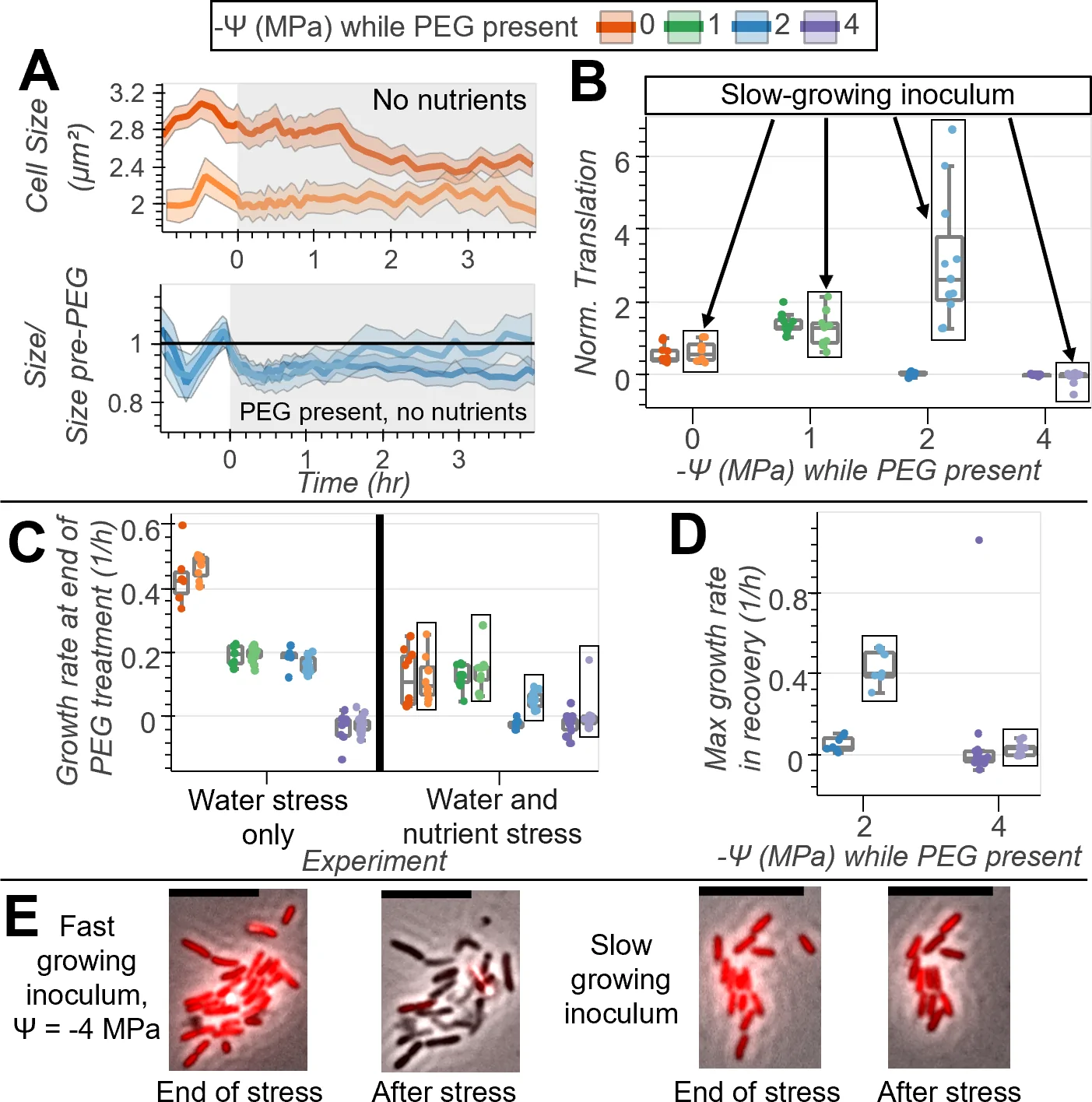
Cellular responses to the simultaneous removal of water and nutrients vary with growth rate prior to stress onset. (**A**, top) Mean size of reporter cells that experienced only nutrient stress beginning at *t* = 0 h. Ranges show 95% confidence intervals. The lighter colored line shows means from the replicate experiment with a slower-growing inoculum. During PEG exposure (gray shading), there was an average of 84 cells present for the replicate with a faster-growing inoculum (replicate 1, dark colored line) and 33 cells present for the replicate with a slower-growing inoculum (replicate 2). (**A**, bottom). Mean normalized size of reporter cells for which nutrients were removed and the water potential was reduced to −Ψ = 2 MPa at *t* = 0 h. Cell sizes are normalized to the mean value in the 30 min leading up to PEG addition. During PEG exposure, there was an average of 97 cells present for replicate 1 and 43 cells present for replicate 2. Complete cell counts for the entire time series are in Fig. S6. (**B**) The ratio of total estimated mNeonGreen to mApple translation calculated using a model that accounts for photobleaching and the dilution of fluorescent proteins by growth (see Materials and Methods). Points represent microcolonies, and points in boxes represent microcolonies that originated from the slower-growing inoculum. Higher values of this ratio correspond to greater overall NAGGN gene expression. (**C**) Mean growth rate in the last hour of PEG exposure, comparing the experiments with water stress only (left) and experiments where water and nutrients were simultaneously removed (right). (**D**) The maximum growth rate sustained for at least 0.5 h for microcolonies in the −Ψ = 2 and 4 MPa treatments after PEG was removed and nutrients were restored. (**E**) Images of reporter cells at two different time points: (i) after 4 h of simultaneous PEG exposure (−Ψ = 4 MPa) and nutrient limitation and (ii) immediately after PEG was removed and nutrients were restored for recovery. Cells on the left originated from the fast-growing inoculum and cells on the right originated from the slow-growing inoculum. The red overlay shows constitutive mApple fluorescence. The same intensity scale was used for all images, and scale bars are 10 µm.

**Fig 6:**
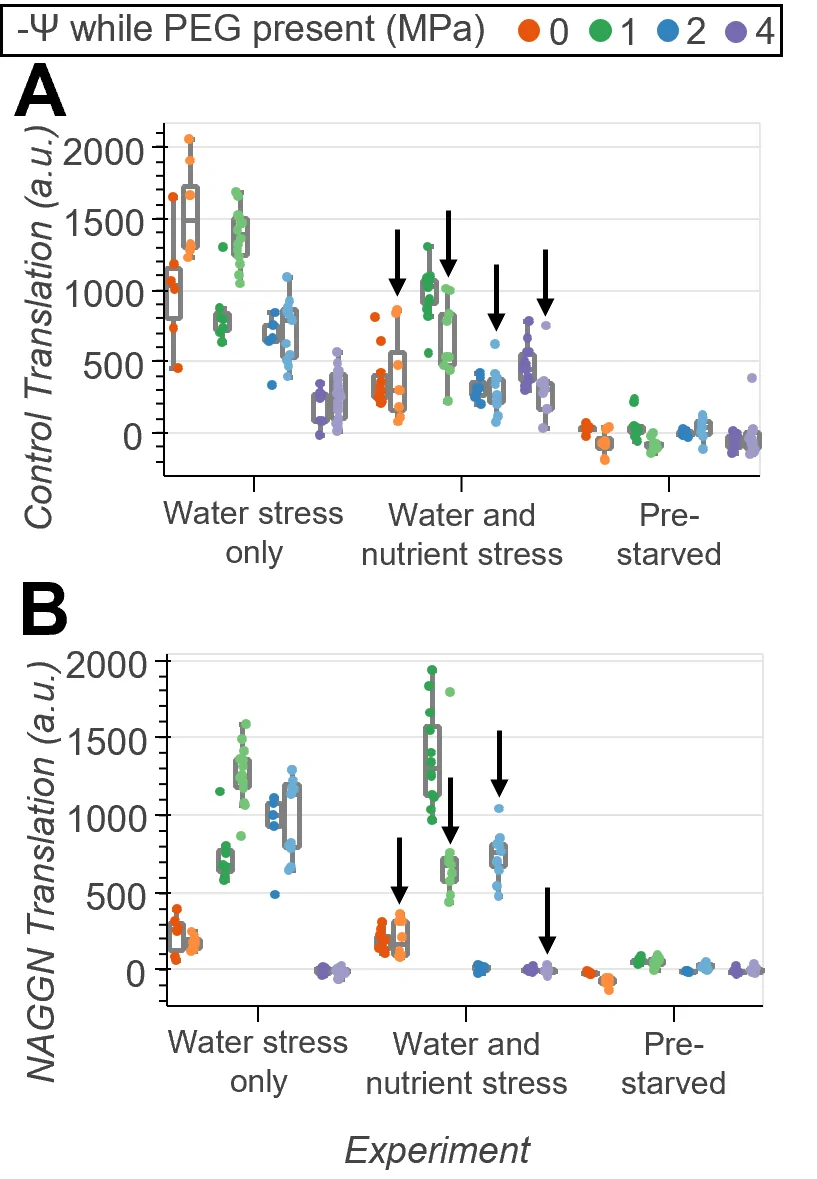
Comparing integrated fluorescent protein translation between nutrient and water stress treatments. (**A**) Total amount of mApple translation that occurred during PEG exposure across all experiments. Colors distinguish between water potentials. Lighter and darker shades represent different experimental replicates. For the experiments where water and nutrient stress began simultaneously (here labeled “Water and nutrient stress”), arrows point to the biological replicate that began with a slower-growing inoculum. As these values were computed from fluorescence data (see Materials and Methods), they are expressed in arbitrary units. Points correspond to the microcolonies. (**B**) Total amount of mNeonGreen translation during the PEG treatments under control of the P_ggnA_ promoter.

First, we examined cellular responses to nutrient removal in the absence of water stress (Ψ = 0 MPa). In both inocula, growth rates decreased to similar levels upon nutrient removal, as expected under nutrient deprivation (red points in Fig. 4C, right). In the faster-growing inoculum, cells were larger when entering the starvation treatment (Fig. S3C) and shrank in size after 2 h of nutrient deprivation (dark red line in Fig. 4A, top), consistent with the expected correlation between bacterial cell size and growth rate (27). Eventually, these cells were only slightly larger than the cells from the slower-growing inoculum, which entered the starvation treatment at a smaller size (faint red line in Fig. 4A, top). Cells under nutrient starvation made less mApple than when nutrients were never removed, consistent with reduced protein synthesis under nutrient limitation (0 MPa data points in Fig. 6A), with similar mApple translation between inocula.

There were also only modest differences between inocula for the Ψ = −1 MPa treatment. Similar to the response when nutrients were maintained throughout water stress, cells did not noticeably shrink immediately after PEG addition despite the hyperosmotic shock (Fig. S3D). Growth rate decreased when PEG was added and nutrients were removed, but this growth rate matched values observed under nutrient removal only (compare green and red points in Fig. 4C, right). In other words, water stress did not further reduce growth rates under these conditions, even though water stress sharply decreased the growth rate in the previous experiments when nutrients were not removed (Fig. 4C, left). The cells expressed NAGGN biosynthesis genes at high levels; in both replicates, normalized mNeonGreen translation during PEG exposure matched levels seen when nutrients were never removed (compare Fig. 4B and Fig. 3C). This response was again rapid in onset but transient, with the normalized fluorescence decreasing rapidly after reaching its peak in 1 h (Fig. S3E and F). Differences in prior growth conditions had only subtle effects on *P. synxantha’*s ability to withstand nutrient stress occurring in isolation or in combination with mild water stress.

However, more extreme water stress amplified the differences between the inocula. The faster-growing cells shrank by about 10% immediately after the water potential was lowered to Ψ = −2 MPa (dark blue line in Fig. 4A, bottom), a similar extent to what we observed when nutrients were present (Fig. S2B). These cells could not recover their original size, stopped growing completely (dark blue points in Fig. 4C, right), and did not resume growth even after PEG was removed and nutrients were restored (dark blue points in Fig. 4D). Even though the estimated amount of mApple synthesis during PEG exposure was similar for both inocula and matched the levels seen with no PEG (blue points in Fig. 6A, water and nutrient stress experiment), there was no detectable NAGGN gene expression by cells from the faster-growing inoculum (dark blue points in Fig. 4B and Fig. S3E). Water stress was better tolerated by the slower-growing cells, which also initially shrank by ~10% but regained their original size within 2 h of PEG exposure (light blue line in Fig. 4A). Growth continued, albeit at a very slow rate (light blue points in Fig. 4C, right), and the growth rate dramatically increased once PEG was removed and nutrients were restored (Fig. 4D). NAGGN gene expression followed similar patterns to what we observed with nutrients present: gene expression at Ψ = −2 MPa was delayed in onset but persisted for longer when compared to the response at Ψ = −1 MPa (Fig. S3F). The ratio of integrated mNeonGreen to mApple translation was also higher than in any other condition we tested (Fig. 4B). Slower-growing cells were better able to withstand and adapt to PEG addition.

We observed similar results for the Ψ = −4 MPa treatment. This water potential was difficult for *P. synxantha* to manage. First, in both replicates, cells shrank (Fig. S3G) to a similar extent as when nutrients were present during water stress (Fig. 3D). Subsequently, they could not grow while PEG was present (Fig. 4C) or resume growth when water and nutrient stress ended (Fig. 4D) and failed to express NAGGN biosynthesis genes (Fig. 4B). However, removing PEG and restoring nutrients caused more cell lysis for the faster-growing inoculum as indicated by a much larger decline in control fluorescence observed at the time of this transition (Fig. S3H, see images in Fig. 4E). Again, the slower-growing inoculum was better able to withstand large and rapid shifts in water potential.

### Response of pre-starved *P. synxantha* cells to water stress

Motivated by our observation that slower-growing cells appeared to have an advantage under water stress, we sought to explore putative connections between prior nutrient limitation and water stress tolerance by pre-starving *P. synxantha* before adding PEG. This experiment approximates the starvation that may precede dehydration in some soils (Fig. 1). Specifically, we deprived *P. synxantha* of nutrients for 16 h prior to PEG addition, predicting that pre-starvation would enhance *P. synxantha*’s tolerance to large shifts in water potential.

**Fig 5:**
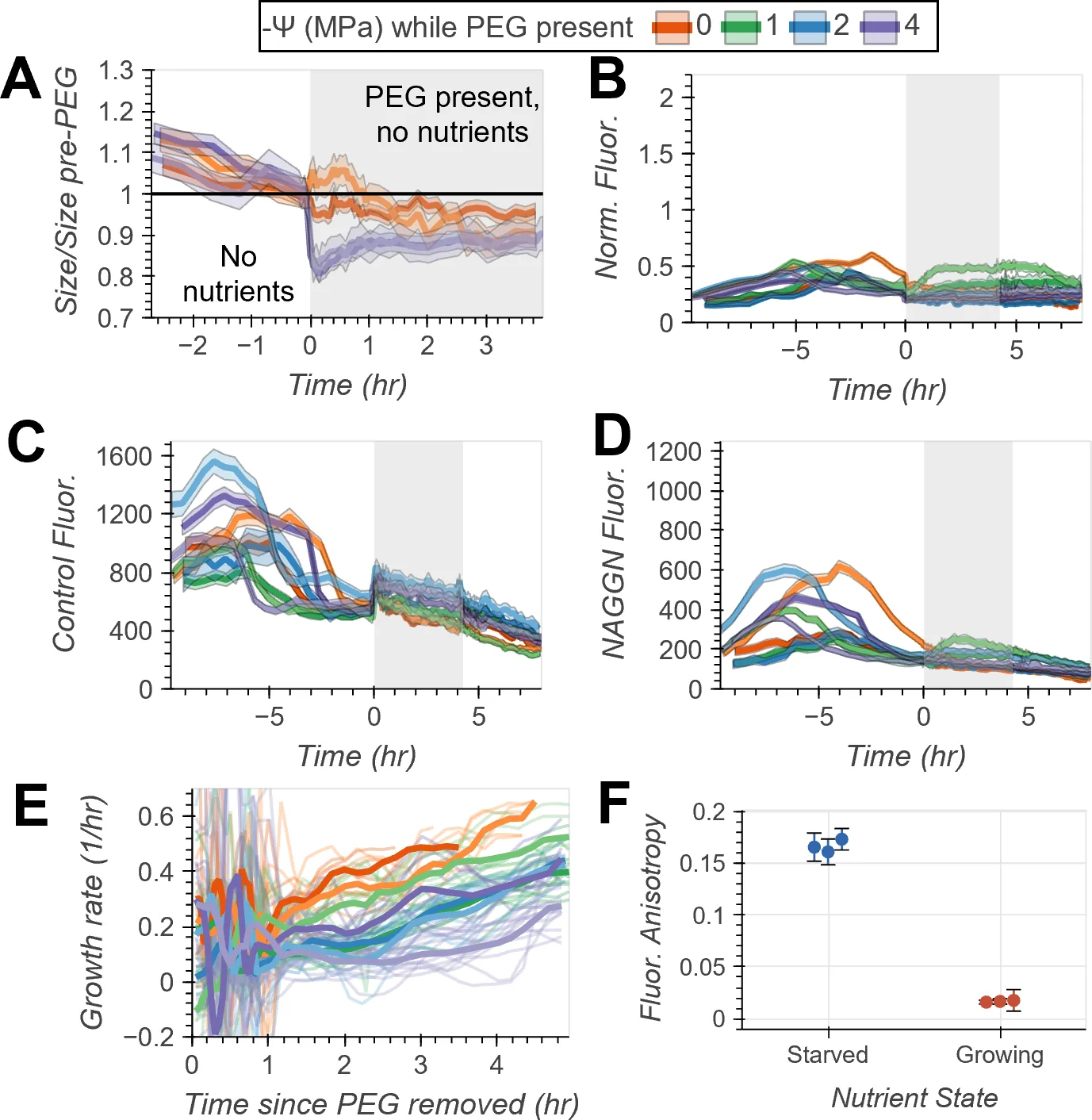
Pre-starved cells can recover from severe water stress without expression of NAGGN biosynthesis genes. (**A**) The normalized size of reporter cells after 16 h of pre-starvation and over the course of 4 h of simultaneous starvation and PEG exposure (gray shading). Lines show the mean cell size at each time point normalized to the mean size in the 30 min leading up to PEG addition. Colors show different water potentials, and the darker and lighter shades distinguish between replicate experiments. Ranges show 95% confidence intervals. During PEG exposure, there was an average of 145–355 cells present for the water potentials shown, with numbers varying between water potentials and replicates. Complete cell counts for the entire time series are in Fig. S6. (**B**) The ratio of NAGGN reporter to constitutive fluorescence throughout the last 10 h of pre-starvation, 4 h of PEG exposure in the absence of nutrients, and 4 h of recovery with water and nutrients restored. Lines show mean normalized fluorescence across all segmentation masks, including those with multiple cells. Ranges show 95% confidence intervals. During PEG exposure, there was an average of 128–469 masks present, varying between water potentials and replicate experiments. Complete mask counts for the entire time series are in Fig. S5. (**C**) Constitutive mApple fluorescence during the same period, with the same number of masks used to compute averages. (**D**) mNeonGreen fluorescence, which peaked at the same time as mApple fluorescence during pre-starvation. (**E**) Mean microcolony growth rates after PEG was removed and nutrients were restored. Bold lines show the mean growth rate across all microcolonies, and faint lines show growth rates for individual microcolonies. The number of microcolonies measured at all time points is displayed in Fig. S7. (**F**) Fluorescence anisotropy of 1,6-diphenyl-1,3,5-hexatriene (DPH) membrane dye staining starved or exponential phase cells. Higher values indicate lower membrane fluidity. Points are separate biological replicates, and error bars show the standard error of five technical replicates.

Immediately after PEG addition, lowering the water potential had a similar effect on the pre-starved cells as it did in the other nutrient conditions. Cells initially shrank by a similar amount as when nutrients were present (compare Fig. 5A and Fig. 3D). In our previous experiments, cells could not permanently recover any of their lost volume when shifted to a water potential of Ψ = −4 MPa. However, with prior starvation, cells shifted to this water potential rebounded slightly in size within 1 h of PEG addition (purple lines in Fig. 5A). It is unclear how this recovery occurred, as there was no detectable NAGGN gene expression following PEG addition (Fig. 5B and Fig. 6B). New gene expression may have simply been impossible during this period, as overall mApple translation was negligible (Fig. 6A), and the control fluorescence even decreased while PEG was present (Fig. 5C). Prior to PEG addition, the mNeonGreen fluorescence did increase, peaking after cells had been in the starvation medium for roughly 9–12 h (*t* = −7 to −4 h in Fig. 5D). It was difficult to estimate total translation from fluorescence data during this period because our method requires growth rate data to account for fluorescent protein dilution. The growth rate, which by visual inspection appeared to be decreasing, could not reliably be estimated because cells began stacking on top of each other and later detached from the Petri dish surface in large numbers as starvation became severe (Fig. S4A). However, the mNeonGreen fluorescence peak was highly variable between water potentials and biological replicates and coincided almost exactly with a peak in mApple fluorescence (Fig. 5C and D). Therefore, the normalized fluorescence changed very little (Fig. 5B), implying that the observed increase in mNeonGreen fluorescence was not driven by the upregulation of NAGGN biosynthesis genes. Instead, changes that would affect both the mApple and mNeonGreen fluorescence in tandem likely explain this peak. Decreases in the growth rate during starvation would decrease the rate at which fluorescent proteins are diluted, and fluorescence can appear brighter because of vertical cell stacking. Consequently, we concluded that the extent of NAGGN biosynthesis by the starving cells was insignificant, so this osmolyte could not have contributed to *P. synxantha*’s survival through subsequent water stress and re-wetting.

Even though the pre-starved cells could not mount a transcriptional response to any decrease in water potential, they were better able to survive the transition back to unstressed conditions. The Ψ = −4 MPa cells, which lysed after removing PEG in the other treatments, remained intact (Fig. S4B) and showed no acute loss of fluorescence once PEG was removed and nutrients were restored (Fig. 5C). These cells were later able to resume growth, a testament to their enhanced resilience (Fig. 5E). This recovery was heterogeneous, as some cells began to elongate and divide while others recovered little in size (see increased variance in cell size distributions in Fig. S4C and images in Fig. S4B). Still, the overall trends in growth and lysis demonstrate that prior stress exposure hardened *P. synxantha* cells against fluctuations in water potential, suggesting a role for starvation adaptation in bacterial survival through soil drying and rewetting cycles.

Because prior starvation prevented cell lysis upon rewetting, we tested the hypothesis that pre-starved cells were more desiccation tolerant due to changes in their membranes. This possibility follows from previous research indicating that starvation alters membrane lipid composition (28), with uncertain consequences for membrane fluidity (43, 44), a key variable linked to desiccation tolerance (30). We compared the membrane fluidity of starved and exponential phase *P. synxantha* cells using 1,6-diphenyl-1,3,5-hexatriene (DPH), a lipophilic fluorescent dye. When excited with polarized light, the anisotropy of this dye’s emission measures its rotational diffusivity inside the lipid bilayer. DPH fluorescence anisotropy therefore provides a qualitative measure of membrane microviscosity or membrane fluidity (43). After pre-growing wild-type *P. synxantha* cells to exponential phase, we washed these cells via centrifugation in starvation or nutrient replete media. We then added DPH to the nutrient replete cells and measured fluorescence anisotropy for an exponential phase control. We allowed the other cells to starve for 16 h, as was done in the microscopy experiments, and repeated the same DPH measurement. The starved cells had a much higher fluorescence anisotropy than their exponential phase counterparts, indicating that membrane rigidification accompanies starvation in *P. synxantha* (Fig. 5F) and positively correlates with water stress tolerance in this organism.

## DISCUSSION

In this study, we investigated how the model soil bacterium *P. synxantha* responds to desiccation dynamics by (co)-varying the level of water stress and nutrient deprivation it experienced. This combination of stressors reflects the reality in soil environments that desiccation both deprives cells of water and restricts nutrient transport. We used high-resolution time-lapse microscopy to broadly survey *P. synxantha*’s response to separate or combined water and nutrient stress, enabling us to monitor growth, NAGGN biosynthesis gene expression, general protein synthesis, single-cell morphology, and the timing and mechanisms of cell death along different drying trajectories. We found that NAGGN gene expression occurred immediately after the onset of water stress if nutrients were available or if water stress was not too severe. Excessive water stress and starvation both prevented NAGGN gene expression, but prior starvation prevented lysis and membrane permeabilization upon rewetting. Our observations suggest that the order at which nutrient and water deprivation occur in soil is physiologically significant.

If starvation generally precedes water limitation in soil, our data support its role as a powerful adaptive mechanism whereby soil bacteria withstand desiccation. As illustrated in Fig. 1, effective solute diffusivity can be orders of magnitude lower in soil than in aqueous solutions even immediately after rain events that do not fully saturate the soil. Solute diffusivity continues to decline as soil dries, even as the water potential remains wetter than our culture medium without PEG added. In a study comparing nitrification rates in soil slurries and intact soil, nitrification was reduced at mild water potentials in soil primarily because of restricted solute transport (45). These and other findings have inspired attempts to model soil respiration solely as a function of solute diffusivity (46). However, other models suggest that the physiological effects of water stress may affect bacteria before they face restricted nutrient transport (47). The heterogeneity of the soil matrix and the nonlinear relationship between microbial activity and moisture content complicate efforts to understand microbial stress sensitivity using bulk-averaged data (48), motivating our reductionist, single-cell visualization approach. Further research is needed to understand the extent to which our laboratory experiments map to soil microbial behavior at the pore scale along a dynamic drying trajectory in natural environments.

Though our results point to a strong role for starvation in promoting desiccation tolerance as desiccation intensifies, they do not rule out potential contributions from osmolyte synthesis. While we did not detect NAGGN gene expression in response to starvation or to water stress in previously starved cells, other osmolytes made by soil microorganisms (such as trehalose and amino acid osmolytes) may confer greater desiccation protection (19, 49, 50). However, when these osmolytes were measured in the field, their concentrations were too low to balance the water potential of the soil and prevent cytoplasmic water loss; instead, they may be protecting biomolecules from desiccation damage at the molecular level (19, 49). For instance, trehalose can protect membranes via direct hydrogen bonding with phospholipid headgroups, maintaining lipid spacing and preventing a destructive phase transition from a liquid crystal to a gel phase upon dehydration (51, 52). Perhaps NAGGN is needed at only very low concentrations to confer similar protection, or other osmolytes are more important in starved cells. Either way, the protective benefits of starvation are not limited to any starvation-induced osmolyte synthesis, as supported by our results and studies with other osmolytes. Starved yeast cells lacking trehalose biosynthesis genes still survive short-term desiccation at a rate 1,000-fold higher than for dividing cells (53), and *E. coli* stationary phase cells exhibit elevated desiccation tolerance despite a lack of trehalose accumulation (54). Our experiments reach beyond these results to characterize how single-cell morphology, growth, and gene expression respond to combined nutrient and water stress, providing temporally resolved information that extends prior measurements of cell viability following a period of desiccation.

Because we chose to study desiccation via time-lapse microscopy, we observed how and when cells died along different drying trajectories. Seeing that starvation prevented cell lysis upon rewetting, we were motivated to study how starvation affected the cell membrane. We observed a large decrease in membrane fluidity following starvation in *P. synxantha*, consistent with some, but not all, prior measurements in bacteria (43, 44, 55, 56). Membranes passively rigidify under desiccation as cell shrinkage causes lipid packing density to increase (52). Under mild dehydration, this can be destructive as membranes unevenly transition from the liquid crystal to the gel phase, causing leakage of cytoplasmic contents upon rewetting (30). When cells were not starved prior to water stress, we observed leakage of cytoplasmic fluorescent proteins or outright cell lysis following PEG removal. It was surprising then, that membrane rigidification under prior starvation was correlated with reduced leakage and lysis following rewetting. Under more extreme water stress, membranes in the liquid crystal phase can instead transition into a hexagonal phase in which inverted micelles are favored over lipid bilayers, a catastrophic event for a cell (30). This transition is facilitated by cis-unsaturated fatty acids, which are converted to trans-unsaturated fatty acids in the membranes of *Pseudomonas putida* following PEG addition to −1 MPa. Because cis-unsaturated fatty acids fluidize the membrane, this adjustment would favor membrane rigidification (29). In our experiments, starvation likely allowed *P. synxantha* to adjust its membrane composition prior to water limitation, preventing membrane damage during desiccation and death upon rewetting. Future studies of membrane remodeling over the course of drying would be informative.

In summary, our work shows that a transition to low growth rate prior to the onset of water stress can promote desiccation tolerance, a stress that fundamentally involves co-limitation by water and nutrients. In soil, this transition is likely to occur through starvation, but the particular physicochemical properties that make starved cells more desiccation tolerant are not fully understood. Identifying these properties would improve our understanding of dormant cell biology and is a priority for future research.

## MATERIALS AND METHODS

### Strains and culture conditions

All strains were derived from *Pseudomonas synxantha* 2-79. This organism was isolated from a dryland wheat field in eastern Washington (57), where it defends wheat roots from fungal infection by producing antifungal phenazine compounds (58). Across the region, its relative abundance is highest in non-irrigated, drier sites (59). Unless otherwise indicated, cells were grown at 30°C with shaking in a defined medium (hereafter “SR”) intended to mimic the stoichiometry of soil microbial biomass (see composition in Table S2). The medium was also designed to have a low osmolarity and a pH that matched that of 1/2-21C, another low osmoticum medium used in prior studies of *Pseudomonas* osmotic stress response (c.f. references 29, 35, 39). For experimental treatments intended to impose nutrient starvation, we removed all sources of carbon (other than PEG), nitrogen, and phosphorus from SR and added more NaCl and MOPS buffer to match the osmolarity and ionic strength of SR, creating the medium SRM. Unless otherwise indicated, polyethylene glycol 1000 (PEG) was used to impose water stress at concentrations of 140, 209, and 300 g/L, corresponding to water potentials of Ψ = −1.26, −2.00, and −3.65 MPa as measured by a WP4C dew point psychrometer. In the figures, these treatments are rounded to approximate water potentials of Ψ = −1, −2, and −4 MPa, respectively. Cells in the treatment labeled 0 MPa never received PEG. Tetracycline was used at a 15 µg mL^−1^ concentration (Tet15) when required for selection or during all experiments with the reporter strain to maintain the reporter plasmid. *Escherichia coli* DH10*β* was used as a cloning host strain, and gentamicin was used at 20 µg mL^−1^ (Gen20) with this strain for selection.

### RNA sequencing

We picked two colonies of wild-type *P. synxantha* from an LB agar plate and grew them overnight in separate 5 mL volumes of SR. Then, we diluted these cultures to OD 0.1 and grew them to OD 0.4–0.6, or mid-exponential phase. We split each culture into halves, one that was washed twice with SR and another that was washed with SR augmented with 280 g/L PEG 8000 (centrifugation done at 6,800 × *g* for 5 min). After returning the cultures to 30°C with shaking for 30 min, we immediately pelleted the cells and performed RNA extractions as described in reference 60. We verified RNA integrity using an Agilent Bioanalyzer and performed rRNA depletion using a Ribo-Zero Plus rRNA depletion kit (Illumina). Then, libraries were prepared using a NEBNext Ultra II RNA-seq kit (New England Biolabs), and sequencing was done on an Illumina HiSeq 2500 with a 2 × 50 bp configuration. Finally, we used Bowtie 2 (61) to align reads and featureCounts (62) to count gene expression. The R package DESeq2 (63) was used to compute the reported gene expression fold changes and *P* values. Fold changes and computed *P* values for all genes are available in Data set S1.

### Construction and validation of NAGGN biosynthesis reporter

After identifying NAGGN biosynthesis as a relevant target for further analysis, we designed a transcriptional reporter for the expression of the NAGGN biosynthesis genes. First, we used PCR and Gibson assembly to generate a plasmid containing the promoter sequence upstream of *ggnA* identified by reference 40, a synthetic ribosome binding site (RBS) (64), and the gene for the fluorescent protein mNeonGreen (65). This plasmid was built on a modified pPROBETT backbone (66), adjusted to remove redundant terminators included in the original backbone sequence and containing an origin of replication that is functional in *Pseudomonas* (sequence in Data set S3). We electroporated the plasmid directly into competent *P. synxantha* cells prepared according to reference 67. After 2 h of recovery in Luria broth (LB) at 30°C, we plated transformants on LB Tet15 plates and allowed colonies to grow at 30°C overnight. Then, a subset of colonies was picked for sequence verification. Plasmids were purified using a New England Biolabs Miniprep kit from cultures derived from these colonies and sequenced by Plasmidsaurus. A plasmid with the correct sequence was stored at −20°C in ddH_2_O for future cloning.

Because osmotic stress alters the overall capacity of a cell to synthesize proteins (68), we also developed a constitutively fluorescent strain to account for this effect when considering changes in reporter fluorescence intensity. We chose the constitutive promoter for the neomycin phosphotransferase on the Tn5 transposon (P_nptII_), as this promoter was used as a positive control for gene expression in a prior study of *Pseudomonas* water stress response (39). We first attempted to add P_nptII_, another synthetic RBS, and the gene for the fluorescent protein mApple (69) to the plasmid harboring our NAGGN reporter construct. However, transforming this plasmid into *P. synxantha* significantly reduced its growth rate, so we instead inserted a single copy of the constitutive reporter construct into a neutral site on *P. synxantha*’s chromosome. We used Gibson assembly to insert the construct into the pJM220 backbone (70) and transformed this plasmid into electrocompetent *E. coli* DH10*β* prepared using standard methods (71). After plasmid purification and sequence verification, we then transformed this plasmid and the helper plasmid pTNS1 (72) into electrocompetent *P. synxantha*. pTNS1 contains an integrase that catalyzed insertion of our constitutive reporter construct into the single *att*Tn7 site on *P. synxantha*’s chromosome as described in (72); pTNS1 and the pJM220 reporter plasmid are non-replicating in *P. synxantha* and so were not retained after outgrowth. A similar approach was used to generate a strain with mNeonGreen constitutively expressed under the control of the P_A1/04/03_ promoter (73) for photobleaching characterization. We also attempted to insert our NAGGN reporter construct into the *att*Tn7 site following the same strategy, but the level of expression driven by the NAGGN promoter was too weak to be detected when it was present only as a single copy. Thus, we transformed the constitutively mApple-producing red fluorescent strain with the NAGGN reporter plasmid described above, producing the “dual reporter” strain used for all subsequent experiments. Strains, plasmids, and primers for all cloning are listed in Table S3.

Finally, we conducted a plate reader experiment to verify that our reporter strain responds to water stress. We grew the strain overnight to stationary phase in SR at 30°C before diluting this culture to OD 0.1 and regrowing it to mid-exponential phase (OD 0.4–0.6). Then, we distributed this culture in 200 µL aliquots in a 96-well microtiter plate and recorded the OD_600_, red fluorescence (excitation = 561 nm, emission = 610 nm), and green fluorescence (excitation = 485 nm, emission = 535 nm) every 20 min for 1 h using a Tecan Spark 10 M plate reader to establish baseline activity. Incubations were done with shaking at 30°C. The first hour in Fig. 2B shows data from this incubation used to establish a baseline level of reporter fluorescence. Separately, we prepared a plate with wells half filled with either SR or SR augmented with 400 g/L PEG 1000, roughly twice the concentration required to achieve a final water potential of −Ψ = 2 MPa. We transferred 100 µL of the cultures from the first plate into the plate with the PEG-amended media, mixed thoroughly via pipetting, and placed this new plate into the plate reader to record the response to this hyperosmotic shock. The lines in Fig. 2B after PEG addition show data for the culture diluted 1:1 into SR (Ψ = 0 MPa) or into SR + PEG 1000 (Ψ = −2 MPa).

### Microscopy under water and nutrient stress

We began each water and nutrient stress experiment by preparing a mid-exponential phase culture of the reporter strain as in our reporter tests above. We also prepared a PDMS well plate using a method adapted from reference 41. Briefly, we prepared 20 × 20 mm squares of 2 mm thick PDMS and used a biopsy punch to create four 8 mm diameter wells in the PDMS. Then, we cleaned the PDMS and a 50 mm glass-bottomed petri dish with 70% ethanol, 100% isopropanol, and 70% ethanol sequentially and plasma bonded the PDMS to the petri dish. Immediately after plasma bonding, we added 50 µL of Corning CellTak adhesive solution prepared according to the manufacturer’s instructions to each well. After letting it sit for 20 min and rinsing out the CellTak solution, we diluted the mid-exponential phase culture to OD_600_ = 0.0015 in SR Tet15 and added a 0.5 µL droplet of culture to the center of each well. To limit evaporation, we also added 1 mL of SR with 15 µg L^−1^ tetracycline (SR Tet15) to the space surrounding the PDMS block inside the petri dish. We glued the petri dish to a custom 3-D printed stage insert and installed a lid with heating wires maintained at 32.5°C–33°C using a temperature controller and voltage regulator set at 1.5 V. This temperature setting limited condensation on the lid of the petri dish without preventing growth of the underlying cells. Cells were allowed to settle out of the small droplet for 10 min, during which time they became attached to the CellTak-coated glass at the bottom of each well. We began each experiment by filling the wells with 90–100 µL of SR. At this point, imaging began. All microscopy experiments were done in duplicate.

Images were acquired using a Nikon Eclipse Ti2e microscope equipped with a 4.2 megapixel, 16-bit Orca Flash4.0v3 camera, a Lumencore Spectra X light source, and an Okolab H201 temperature control unit held at 30°C. We used a 40× 0.95 NA Ph2 objective with an additional 1.5× intermediate magnification tube lens activated for acquisition. In addition to phase contrast images, we collected mNeonGreen fluorescence images using 488 nm LED excitation with a 535/40 nm emission filter, and mApple fluorescence images using 561 nm LED excitation with a 620/60 nm emission filter. To ensure an adequate sample size of cells, we selected three fields of view for monitoring from each of the wells. Fields of view (FOVs) were selected to contain a few to a dozen cells with adequate separation so that we could track the activity of microcolonies that developed from parent cells present at the beginning of the experiment. We imaged the cells every 10 min for 6 h before beginning the next stage of the experiment. The purpose of this initial adjustment phase was to allow cells to adapt to the PDMS well plate and microscope environment before proceeding. From here, each experiment proceeded differently according to the type and sequence of imposed stresses; timelines of each experiment are presented in Fig. 2D through F. In the paragraphs below, boldface text corresponds with the names of the experiments used in Fig. 2F and in Fig. 4C and Fig. 6A and B.

For the experiments with **water stress only**, we prepared SR medium augmented with the aforementioned PEG 1000 concentrations to impose water stress, along with a no PEG-added control (Ψ = 0 MPa condition). Then, well-by-well, we removed the existing SR medium, washed once with the target treatment medium, and refilled the well with this treatment medium to start the stress phase of the experiment. Because the petri dish was glued to the stage insert, we could do this without moving the petri dish to continue following the same FOVs selected above. Throughout this process and until an hour thereafter, we imaged every 3 min to capture any rapid osmotically driven changes in cell size that immediately followed PEG addition. We then reduced our imaging frequency to every 10 minutes and continued imaging for 3 more hours, meaning that cells were exposed to PEG-induced water stress for 4 h in total. Finally, we resuscitated our cells by replacing all the media with SR. We again collected images every 3 min for 1 h at the start of resuscitation, before imaging every 10 min for 7 more hours.

For the experiments in which **water and nutrient stress** began at the same time, the experiment proceeded as described above, except that during the water stress, SRM starvation medium augmented with PEG was used.

Finally, for the “**pre-starved**” experiments where nutrients were absent both before and during water stress, we included an additional starvation phase after adjustment and prior to the PEG exposure. After the adjustment phase, we replaced all media with the SRM starvation medium to initiate nutrient stress. During this pre-starvation phase, we imaged every 30 min for 16 h, as changes in cell size and reporter fluorescence were more gradual under this condition. We then replaced the media again with SRM augmented with PEG to initiate water stress, as above, and the rest of the water stress and recovery phases proceeded as outlined in the previous experiments.

### Segmentation and analysis of fluorescence images

To measure cell growth, cell size, and NAGGN biosynthesis reporter activity, we performed image segmentation. During these experiments, cells were initially seeded at low density, but over time they grew into small clusters, which we call microcolonies. We present analysis at the single cell, cell group, and microcolony levels. We limited our analysis to cells that were descendants of a parent cell that was present from the start of the experiment. This gave us information about the reporter’s response to all events in the experiment and limited our focus to cells that were always surface attached and therefore experienced similar conditions. We began by extracting tiff files for each frame from the nd2 files generated by the microscope and registered these images using the MATLAB package OmniSegger (74). Then, we manually selected one to five bounding boxes from each field of view containing a microcolony that developed over the course of the experiment. These boxes exceeded the final area of the microcolony so that its total area could be tracked over time.

After cropping the tiffs, we generated segmentation masks for cells within each microcolony using Omnipose, a deep neural network for image segmentation (75). We used the phase contrast images for this task with Omnipose’s pre-trained model “bact_phase_omni.” Segmenting individual cells was challenging because cell density within the microcolonies was high. We therefore limited our attempts at segmentation to time points when most cells in the microcolony were visibly distinguishable from each other. This is why some traces for reporter fluorescence and growth rates end earlier than others, especially in the milder stress conditions where microcolonies could grow larger and denser. Nonetheless, while some of the generated segmentation masks contained only one cell, many contained multiple. When analyzing our fluorescence data, we calculated the mean background-subtracted fluorescence intensity for all the masks, giving us one fluorescence value per cell or small group of cells. We then plotted the mNeonGreen and mApple fluorescence, as well as the ratio between these two values, averaged across all the masks in a given PEG treatment. Ninety-five percent confidence intervals for means were computed using the t distribution unless otherwise indicated. The number of masks used for these calculations at each time point is reported in Fig. S5. Complete code for plotting and image analysis, as well as a list of required Python packages, are available at https://github.com/jkwiecinski0/NAGGNImageAnalysis (Software S1).

### Measuring cell size

To estimate cell size, we needed a method to filter for masks that contained only an individual cell. To do this, we separated each experiment into different stages where there were large visible differences in cell morphology. Though the exact definitions of these stages differed between experiments (see notebooks/filter.ipynb and src/mask_filter.py in Software S1), the Ψ = −4 MPa cells were almost always analyzed separately from the other populations. Data from the adjustment period before PEG addition was also separated from later time points. Then, from each stage, we randomly sampled 100 masks and recorded the number of cells that fell within each mask. Next, we computed several morphological parameters, including the length, width, and area occupied by the masks, as well as the minimum curvature of the mask perimeters. We used principal component analysis to aggregate all of this information into two parameters and applied constrained optimization to find bounds on the principal components that effectively selected for masks containing only one cell. In our optimization approach, we sought to find bounds that maximized the fraction of the selected masks that contained only one cell. To ensure that the selected masks were still representative of the data, we required that our bounds included 95% of the masks from our random sample containing only one cell. Though these criteria could not always be met, the fraction of masks with one cell and the fraction of one-cell masks contained within our selected bounds were always greater than 84%. Values of the relevant principal components were then computed for the entire data set and masks with more than one cell were filtered out using the optimized bounds. Figure S6 shows how many cells were left over at each time point after we performed this filtering. The means and confidence intervals for normalized size are derived from the filtered masks.

### Measuring microcolony growth rates

Segmentation quality was insufficient to track individual cells and compute single-cell growth rates. However, it was possible to track microcolonies of cells that grew from individuals at the start of each experiment and to measure their area over time. It was rare for cells to suddenly appear from elsewhere, so we did not account for the effect of these infrequent events to inflate the measured microcolony area. Conversely, several cells did not remain adhered to the bottom of the wells, so it was necessary to account for cell detachment by first detecting when cells left the field of view. We could detect these events as large, stepwise decreases in the area of the microcolony between adjacent frames and correct for them as needed. Specifically, we took the difference between images containing segmentation masks at adjacent time points and identified objects that had a length and width exceeding minimal values for a cell. The areas of these objects were signed, with negative values if a cell-shaped object disappeared and positive values if an object appeared at a given location. If the sum of these areas was less than −100 pixels, roughly the minimum size of a cell, we concluded that cell(s) had in fact left the field of view. Taking this sum accounted for cases where cells simply moved instead of detaching. Next, we simulated what the total microcolony area would have been had the cells remained and continued growing. Define the time-varying specific growth rate µ(*t*) of the departing cells as


(1) $$\mu (t)\equiv \frac{1}{A_{l}}\frac{dA_{l}}{dt}=\frac{d\ln A_{l}}{dt},$$

where *A_l_* is the area occupied by cells that left the field of view. Solving for *A_l_* gives


(2) $$A_{l}(t)=A_{l}(t_{l})\exp\left(\int_{t_{l}}^{t}\mu (t)\;dt\right),$$

in which *t_l_* is the time when the cells left. We assumed that the departing cells would have grown at the same rate as the cells that remained. In other words,


(3) $$\mu (t)=\frac{d\ln A_{c}}{dt},$$

where *A_c_* is the measured area of the microcolony. We approximated this derivative using a first-order forward difference. This derivative was inaccurate at the time of and immediately before any cell departure event. We replaced the value of the derivative at these times with the value closest in time that was unaffected by a departure. Then, accounting for all cell departures, the corrected microcolony area at time *t* was


(4) $$A^{t}=A_{c}^{t}+\sum_{m}\left[A_{m}^{t_{l,m}}\exp\left(\sum_{t_{l,m}}^{t}\mu^{t}\Delta t^{t}\right)\right].$$

Here, the $A_{m}^{t_{l,m}}$ are the areas of all cells that departed at times *t_l,m_* during the experiment. The measured microcolony areas and growth rates at time *t* are $A_{c}^{t}$ and µ*^t^*, respectively. The time step ∆*t^t^* refers to the time between image acquisitions at time *t*. Values of *A^t^* are plotted in Fig. S8. For the Ψ = 0 MPa treatment in panel A (nutrients present throughout, PEG never added), the corrected area increased exponentially as expected for microbial growth. Specific growth rates presented in the Results were computed by smoothing the corrected growth curves using locally weighted scatterplot smoothing and approximating the derivative as a first-order forward difference. Smoothing was done separately for different experimental stages (i.e., adjustment, starvation, PEG stress, and recovery) to prevent smoothing over sudden changes in growth rate that occurred due to these transitions.

Any lysis event that results in a cell disappearing from view would also be detected using our approach. However, significant lysis was restricted to the Ψ = −4 MPa treatment and occurred only after PEG was removed at the end of the experiments. Growth of the remaining cells was essentially zero, so at worst, our estimates would miss cell death and indicate static biomass under these conditions. Growth estimation code is included in src/growth.py in Software S1.

### Modeling total fluorophore translation

To compare the extent of gene expression between treatments when PEG was present, we developed an integrative measure of reporter activity that accounted for several processes beyond fluorescent protein translation that affect fluorescence. Because mApple and mNeonGreen do not fluoresce until they have matured, we considered two pools of either protein: an invisible pool P and a fluorescent pool F. Light exposure also bleaches fluorescent proteins, causing their fluorescence to decrease over time. Finally, growth dilutes all proteins over a larger volume, decreasing their concentration. Assuming first-order maturation and bleaching kinetics, the cytoplasmic concentrations of P and F vary according to these dynamics:


(5) $$\frac{dP}{dt}=r-k_{m}P-\mu (t)P,$$

(6) $$\frac{dF}{dt}=k_{m}P-\mu (t)F-k_{b}b(t)F.$$

Here, *k_m_* is the maturation rate of protein P, µ(*t*) is the growth rate, and *k_b_* is the photobleaching rate of the matured, fluorescent protein F. The function *b*(*t*) is a sum of rectangular pulses with amplitude 1 and width equal to image exposure time beginning at each time when an image was taken. Finally, *r* is the translation rate, the quantity we aim to calculate, which depends on fluorescent protein gene transcription along with cellular translation capacity and other factors. We measured the cytoplasmic concentration of F in arbitrary fluorescence intensity units, so these units also apply to our estimated concentration of invisible protein P. We measured *k_b_* by placing exponential phase reporter cells in our PDMS well plates filled with SR media. Then, we took 200 images of the cells in rapid succession to induce photobleaching before new translation, protein maturation, or growth could occur. Finally, we estimated *k_b_* by fitting an exponential function to mApple and mNeonGreen fluorescence plotted against total excitation light exposure time (Fig. S9). This value was 0.0180 ± 0.0001 (SE) s^−1^ for mApple and 0.0199 ± 0.0001 (SE) s^−1^ for mNeonGreen. Values of *k_m_* for mApple (1.4 h^−1^) and mNeonGreen (4.2 h^−1^) were taken from fpbase (76), and µ(*t*) was calculated as described above. For the pre-starved experiments, growth rates could only be calculated after PEG was removed and nutrients were restored, so we assumed a growth rate of zero. We estimated the concentration of P at time *t* from F by discretizing equation 6 and solving for P*_t_*:


(7) $$P_{t}=\left(\frac{1}{k_{m}}\right)\left(\frac{F_{t+1}-F_{t}+t_{b}k_{b}F_{t}}{\Delta t}+\mu_{t}F_{t}\right).$$

Here, *t_b_* is the exposure time, or duration of photobleaching, that occurs at each time point when an image is collected, and ∆*t* is the time between images. Microcolony-averaged values of F were smoothed via locally weighted scatterplot smoothing before they were substituted into this equation. The translation rate *r* was then computed by discretizing Equation 5 and substituting the estimated values of P*_t_* into Equation 5:


(8) $$r=\frac{P_{t+1}-P_{t}}{\Delta t}+k_{m}P_{t}.$$

The total translation values for mApple and mNeonGreen that we report in Fig. 6 were computed by integrating our estimates of *r* for each fluorophore over the time PEG was present. These values were slightly negative for some of the more stressful treatments, perhaps due to fluorophore leakage, degradation, or other processes not included in our model. For this reason, we did not compute the ratio of integrated mNeonGreen to mApple fluorescence for the pre-starved cells, as both the numerator and denominator were near zero. This model is implemented in src/translation.py in Software S1.

### Measuring membrane fluidity

To measure membrane fluidity in starved and exponential phase cells, we grew triplicate 5 mL cultures of wild-type *P. synxantha* overnight in SR medium. These cultures were inoculated from separate colonies on an LB agar plate. Then, we diluted these cultures to OD 0.1 and grew them to OD 0.4–0.6 as was done for the microscopy experiments. Next, we pelleted the cultures at 5,250 × *g* for 5 min and discarded the supernatant. We diluted half of the pellets to OD 0.1 in 5 mL SRM medium and incubated them with shaking at 30°C for 16 h of starvation. We diluted the other half of the pellets to OD 0.1 in fresh SR medium and immediately treated these cells with DPH, a membrane dye used to measure membrane fluidity. We added 4 µL of 1 mM DPH dissolved in DMSO to 400 µL of the exponential phase cultures, incubated this solution at 30°C in the dark for 30 min, and measured fluorescence anisotropy with a HORIBA Instruments Fluorolog fluorometer (see reference 77 for details). We collected five repeated anisotropy measurements, or technical replicates, per biological replicate. We repeated these steps for the starved cells after the starvation period was complete.

## Data Availability

RNA sequencing raw reads are available at https://www.ncbi.nlm.nih.gov/bioproject/PRJNA1397269 (Data set S2).

## SUPPLEMENTAL MATERIAL

Data set S1 mbio.01903-26-s0001.txt RNASeq results.

Data set S3 mbio.01903-26-s0002.txt Annotated sequence of reporter plasmid.

Supplemental Material mbio.01903-26-s0003.pdf Figures S1 to S9, Tables S1 and S2, and captions for other supplemental files.

Table S3 mbio.01903-26-s0004.xlsx Strains.

ASM does not own the copyrights to Supplemental Material that may be linked to, or accessed through, an article. The authors have granted ASM a non-exclusive, world-wide license to publish the Supplemental Material files. Please contact the corresponding author directly for reuse.
